# Estimating fetal cholesterol synthesis rates by cord blood analysis in intrauterine growth restriction and normally grown fetuses

**DOI:** 10.1186/s12944-019-1117-1

**Published:** 2019-10-25

**Authors:** Ulrich Pecks, Verena Bornemann, Anika Klein, Laura Segger, Nicolai Maass, Ibrahim Alkatout, Christel Eckmann-Scholz, Mohamed Elessawy, Dieter Lütjohann

**Affiliations:** 1 Department of Obstetrics and Gynecology, University Hospital of Schleswig-Holstein Campus Kiel, Arnold-Heller-Straße 3, 24105 Kiel, Germany; 2 Institute for Clinical Chemistry and Clinical Pharmacology, University Clinics of Bonn, Bonn, Germany

**Keywords:** IUGR, FGR, Fetal growth rates, Fetal nutrition, Placental sterol transfer rates

## Abstract

**Background:**

Cholesterol is an essential component in human development. In fetuses affected by intrauterine growth restriction (IUGR), fetal blood cholesterol levels are low. Whether this is the result of a reduced materno-fetal cholesterol transport, or due to low fetal de novo synthesis rates, remains a matter of debate. By analyzing cholesterol interbolites and plant sterols we aimed at deeper insights into transplacental cholesterol transport and fetal cholesterol handling in IUGR with potential targets for future therapy. We hypothesized that placental insufficiency results in a diminished cholesterol supply to the fetus.

**Methods:**

Venous umbilical cord sera were sampled post-partum from fetuses delivered between 24 weeks of gestation and at full term. IUGR fetuses were matched to 49 adequate-for-age delivered preterm and term neonates (CTRL) according to gestational age at delivery. Cholesterol was measured by gas chromatography-flame ionization detection using 5a-cholestane as internal standard. Cholesterol precursors and synthesis markers, such as lanosterol, lathosterol, and desmosterol, the absorption markers, 5α-cholestanol and plant sterols, such as campesterol and sitosterol, as well as enzymatically oxidized cholesterol metabolites (oxysterols), such as 24S- or 27-hydroxycholesterol, were analyzed by gas chromatography-mass spectrometry, using epicoprostanol as internal standard for the non-cholesterol sterols and deuterium labeled oxysterols for 24S- and 27-hydroxycholesterol.

**Results:**

Mean cholesterol levels were 25% lower in IUGR compared with CTRL (*p* < 0.0001). Lanosterol and lathosterol to cholesterol ratios were similar in IUGR and CTRL. In relation to cholesterol mean, desmosterol, 24S-hydroxycholesterol, and 27-hydroxycholesterol levels were higher by 30.0, 39.1 and 60.7%, respectively, in IUGR compared to CTRL (*p* < 0.0001). Equally, 5α-cholestanol, campesterol, and β-sitosterol to cholesterol ratios were higher in IUGR than in CTRL (17.2%, *p* < 0.004; 33.5%, *p* < 0.002; 29.3%, *p* < 0.021).

**Conclusions:**

Cholesterol deficiency in IUGR is the result of diminished fetal de novo synthesis rates rather than diminished maternal supply. However, increased oxysterol- and phytosterol to cholesterol ratios suggest a lower sterol elimination rate. This is likely caused by a restricted hepatobiliary function. Understanding the fetal cholesterol metabolism is important, not only for neonatal nutrition, but also for the development of strategies to reduce the known risk of future cardiovascular diseases in the IUGR fetus.

## Background

Intrauterine growth restriction (IUGR) is a condition where the fetus does not reach its genetically determined growth potential [1, 2]. Affecting approximately 3–8% of all pregnancies it is a major cause of fetal mortality and morbidity; it is also generally considered an independent risk factor for the development of cardiovascular diseases (CVD) later in life [3–6]. Although its pathogenesis remains enigmatic, factors, such as disturbed blood perfusion of the placenta with events of hypoxia-reperfusion injury, increased oxidative stress, accumulation of oxidized LDL, atherogenic changes, and placental damage, have been suggested to play key roles in its etiology [7–9]. Consequently, the resulting “placental insufficiency” leads to fetal malnutrition. One component that is believed to play a decisive role in cellular growth and functionality - and in fetal development - is cholesterol. Cholesterol is the most important sterol in humans and, besides its function in membrane fluidity, it is also a precursor of bile acids and steroid hormones [10–14].

Various enzymatic defects at different stages of cholesterol biosynthesis have been reported and are linked to abnormal fetal development. The most common inborn error of cholesterol synthesis is the Smith-Lemli-Opitz syndrome (SLOS), a 7-dehydrocholesterol-Δ7-reductase deficiency. Clinically, patients frequently present with structural abnormalities of the brain, the skeleton and the skin, underlining the important role of cholesterol in fetal development [15, 16].

In IUGR, cholesterol concentration in fetal blood is low [17–23]. In particular, the high-density lipoprotein (HDL) fraction, which is the main cholesterol acceptor and dominant lipoprotein in the fetus, is diminished by about 50% when compared to appropriately grown fetuses [17]. The cause (maternal, fetal, or placental origin) of the low fetal cholesterol concentration in IUGR is still a matter of debate. In general, the fetus synthesizes most of its cholesterol de novo, although it has been estimated that 20–50% of the fetal cholesterol originates from the mother and is transferred through the placenta by distinctive transport pathways [11, 16, 24, 25]. At the fetal side, cholesterol is released from the placenta to circulating acceptors, such as apolipoproteins, and native HDL particles [26]. The hypothesis of a relevant transplacental transport of cholesterol from the mother to the fetus is supported by the analysis of plant sterols. As plant sterols cannot be synthesized in humans, their blood concentration is dependent on ingestion or, in case of the fetus, solely on the amount transported from the mother through the placenta. Plant sterols use the same transport mechanisms as cholesterol and can be detected in relevant concentrations in amniotic fluid and umbilical cord blood [11, 27].

In this study, we measured biochemical markers of cholesterol biosynthesis, lanosterol, lathosterol, and desmosterol, in venous umbilical cord sera of IUGR and normally grown fetuses. We also determined 5α-cholestanol and the most prominent plant sterols, campesterol and ß-sitosterol levels, assuming these to be valid markers to estimate materno-fetal cholesterol transport. Finally, we estimated oxysterol concentrations as a means of cholesterol catabolism.

We hypothesized that a diminished fetal cholesterol concentration is caused by a reduced maternal cholesterol supply as a consequence of placental insufficiency and disturbed transport pathways through the placenta.

## Methods

Biomaterial for this case control study was sampled prospectively at the University Hospital of the RWTH Aachen, Germany. Patients were included between March 2008 and March 2012. Prior to the study, a power analysis was performed. Calculating the minimum required sample size on the basis of the fetal HDL cholesterol concentration, choosing a power (1–β-error) of 80% and a level of significance (α-error) below 0.05 for each group, IUGR and control group (CTRL), at least five patients had to be included in each group.

Clinical data were recorded at inclusion and following delivery. Gestational age was calculated by the last menstrual period and verified by first trimester scan documentation. Fetal cord blood was sampled immediately after birth and processed as described [17].

IUGR was diagnosed antenatally, as recently described, and defined in accordance with the guidelines of the American College of Obstetricians and Gynecologists and the German guidelines on IUGR [1, 2, 17] as an estimated fetal weight < 10th percentile, in addition to at least one of the following criteria: (1) deceleration of fetal growth rate > 40th percentile, (2) elevated resistance index in umbilical artery Doppler sonography above the 95th percentile, (3) head-to-abdominal circumference ratio > 95th percentile or (4) amniotic fluid index < 6 cm. Of the IUGR cases, 13 were additionally complicated by preeclampsia as defined by the ISSHP criteria [28]. IUGR was subgrouped into early-onset IUGR (detection and delivery before 34 weeks of gestation) and late-onset IUGR (detection and delivery at 34 weeks of gestation or later). Fetal and neonatal birth weight centiles were determined according to the population-based newborn weight charts [29]. Additionally, customized centiles were calculated by use of the online platform www.gestation.net (Gardosi J, Francis A. Customised weight centile calculator. GROWTH V6.7.6.11 (DE)).

A total of 299 patients giving birth at our University Hospital were enrolled during the study period. Of those, we identified 49 IUGR cases with sufficient serum volume to be used further for the scheduled investigation. Thirty-six of the IUGR fetuses needed mandatory preterm delivery before 37 weeks of gestation (WOG). The IUGR cases were matched with CTRL as closely as possible for maternal age, gestational age, fetal gender, betamethasone administration, maternal smoking habits, and maternal body mass index (BMI). Neonatal weight in the CTRL group was within the 10th and 90th percentiles. Thirty-two of the 49 CTRL fetuses were born preterm before 37 WOG for various reasons (including premature rupture of the membrane, spontaneous onset of labor, and vaginal bleeding). None of the CTRL mothers suffered from hypertension or preeclampsia.

Exclusion criteria were defined as multiple gestation, fetal anomalies, abnormal fetal karyotype, patients with clinical or biochemical signs of infection, positive toxoplasmosis, rubella, cytomegalovirus, herpes simplex, and HIV (TORCH) screening results, maternal diabetes mellitus/gestational diabetes or other severe maternal metabolic disorders, and the patient’s withdrawal from the study. Sample storage times and conditions were equal for all groups.

### Blood sampling, serum generation, and storage of aliquots

Blood samples (up to 4.9 mL each) were taken postnatally from a double-clamped umbilical cord vein using Monovette syringes (Serum 4.9 mL Monovette; Sarstedt, Nümbrecht, Germany). After incubation at room temperature for 15–30 min, samples were centrifuged at 2000 g for 15 min. Serum was aliquoted and stored at − 80 °C.

### Basic serum lipid profiling

Analysis of serum triglycerides (TG), total cholesterol (TC), LDL- and HDL cholesterol was performed by colorimetric enzymatic methods using an automated photometric measuring unit (Roche/Hitachi Modular P800; Roche Diagnostics, Basel, Switzerland) as described [17].

### Cholesterol, non-cholesterol sterols and oxysterols measurements

After thawing, butylated hydroxytoluene as radical scavenger (antioxidant) and 5α-cholestane, epicoprostanol, and deuterium labelled oxysterols were added as internal standards for the quantification of targets, respectively, to 100 μL of plasma. After alkaline hydrolysis, the free sterols and oxysterols were extracted using cyclohexane and silylated to their corresponding (di) trimethylsilyl ethers prior to gas chromatographic (GC) separation. Cholesterol was determined by flame-ionization detection (FID), the non-cholesterol sterols and oxysterols by selected-ion monitoring mass spectrometry (MS-SIM) as previously described in detail [30–32].

### Statistics

nQuery Advisor 7.0 (Janet D. Elashoff (2007), CA, USA) was used for sample size calculation. Data analysis was conducted using the Prism Version 6.0e Software (GraphPad Software Inc., CA, USA). Clinical data are presented as means ±95% CI or percentages. Analytical variables are expressed as median ± 95% CI. A two-tailed Mann-Whitney test was conducted to compare the metric variables of the two groups. Values were adjusted for potential confounders (maternal age, BMI, parity, smoking status, weeks of gestation at delivery, and fetal stress response (umbilical artery pH)) by residual calculation. Subgroup analysis was performed using the Kruskal-Wallis test followed by Dunn’s comparison for multiple testing. Fisher’s exact test was used for categorical data. Correlations were analyzed by Spearman’s correlation coefficient. Values of *p* < 0.05 were regarded as significant.

## Results

### Study population and basic lipid profile

Maternal and neonatal characteristics are summarized in Table 1. Gestational age at inclusion into the study, gestational age at delivery, mode of delivery, and neonatal gender were kept similar. Maternal age, pre-pregnancy BMI, and smoking status during pregnancy differed slightly, but were not significant between the study groups, while we found a significant proportion of patients in the CTRL group to be multiparous compared to the IUGR group. None of the CTRL group and 27% in the IUGR group were hypertensive during pregnancy. Neonatal birth weight, birth weight centiles, and umbilical artery pH were lower in the IUGR group than in the CTRL group. In the IUGR group, mean fetal TC and HDL cholesterol concentrations were 28 and 48% lower than in the CTRL group (Table 1).

**Table 1:** Maternal and neonatal clinical characteristics and lipid concentrations

		CTRL (*n* = 49)		IUGR (*n* = 49)		*p*-value
		Mean or %	95% CI	Mean or %	95% CI	
Maternal age	(y)	32.0	(30.4–33.6)	29.6	(27.7–31.6)	0.065
Maternal height	(m)	1.66	(1.64–1.68)	1.65	(1.64–1.67)	0.654
Maternal weight b. p.	(kg)	63.7	(60.7–66.8)	68.8	(63.8–73.8)	0.085
Maternal BMI b. p.	(kg/m2)	23.2	(22.1–24.3)	25.0	(23.4–26.6)	0.063
Maternal smoking status	(%)	24.5		36.7		0.192
Maternal primiparity	(%)	32.7		67.4		**0.000**
Maternal systolic BP	(mmHg)	117	(114–120)	129	(123–135)	**0.000**
Maternal diastolic BP	(mmHg)	67	(64–69)	76	(72–79)	**0.000**
Maternal hypertension	(%)	0.0		26.6		0.278
Maternal WOG at inclusion	(w)	33.4	(32.1–34.7)	32.6	(31.4–33.8)	0.360
Delivery WOG	(w)	33.9	(32.7–35.1)	33.6	(32.4–34.7)	0.705
Delivery mode: cesarean	(%)	81.6		83.7		0.792
Neonatal gender	(% female)	59.2		57.1		0.861
Neonatal birth weight	(g)	2246	(2000–2492)	1478	(1295–1661)	**0.000**
Neonatal birth weight centile		43.3	(37.9–48.7)	4.2	(3.4–5.0)	**0.000**
Neonatal customized weight centile		41.7	(34.2–49.2)	0.8	(0.2–1.4)	**0.000**
Neonatal umbilical artery pH		7.33	(7.32–7.35)	7.27	(7.23–7.30)	**0.000**
Neonatal 5 min APGAR value		9.2	(8.9–9.5)	8.9	(8.5–9.3)	0.186
Storage time at − 80 °C	(y)	5.32	(5.08–5.56)	4.98	(4.68–5.27)	0.076
Neonatal LDL cholesterol	(mg/dL)	30	(25–34)	20	(16–24)	**0.002**
Neonatal HDL cholesterol	(mg/dL)	31	(29–33)	16	(14–18)	**0.000**
Neonatal total cholesterol	(mg/dL)	71	(66–77)	52	(47–57)	**0.000**
Neonatal triglycerides	(mg/dL)	21	(18–25)	46	(37–54)	**0.000**

Significant *p*-values are in bold

### Cholesterol

**Fig. 1:**
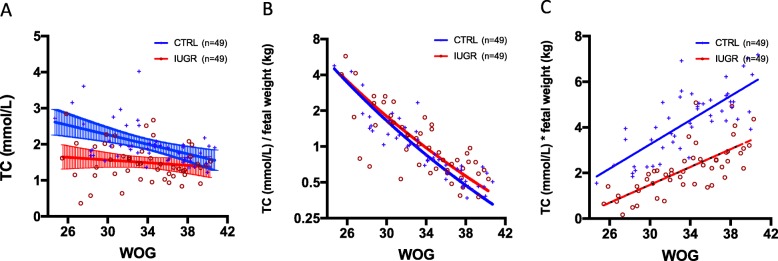
**a** Regression lines along with 95% confidence intervals and individual spots of fetal total cholesterol concentrations (Y-axis) depending on gestational age at delivery (X-axis) are displayed separately for IUGR and CTRL fetuses. Note that blood cholesterol concentrations in IUGR fetuses are lower especially early and before 34 weeks of gestation when compared to CTRL as indicated by the non-overlapping 95% confidence intervals. CTRL: Y = -2.476*X + 161.4, *R*^2^ = 0.2196, *P* = 0.0007. IUGR: Y = -0.8135*X + 85.1, *R*^2^ = 0.0274, *P* = 0.2556. **b** Regression lines and individual spots of total cholesterol concentrations related to neonatal birth weights and depending on gestational age at delivery are displayed separately for IUGR and CTRL fetuses on a logarithmic ordinal scale. Note that fetal cholesterol concentration adjusted to fetal weight in both study groups are similar; both decrease significantly with ongoing gestational age. CTRL: Y = -0.1723*X + 6.988, *R*^2^ = 0.6143, *p* < 0.0001. IUGR: Y = -0.1883*X + 7.643, R^2^ = 0.5226, *p* < 0.0001. **c** Regression lines of assumed fetal body cholesterol accumulation as calculated by blood cholesterol concentration multiplied with birth weight and related to weeks of gestation at delivery are displayed separately for IUGR and CTRL fetuses. CTRL: Y = 0.2656*X - 4.715, *R*^2^ = 0.5709, *p* < 0.0001; IUGR: Y = 0.1955*X - 4.386, *R*^2^ = 0.5214, *p* < 0.0001. Adjusted for gestational age, the demand on cholesterol appears to be twice as high in CTRL compared to IUGR fetuses (*p* < 0.0001)

Cholesterol concentrations measured by GC-FID were virtually identical to those measured with the automated photometric measuring unit Modular P800 (ρ = 0.9371, *p* < 0.0001). Cholesterol concentration in the CTRL group decreases with ongoing gestational age at birth (ρ = − 0.451; *p* = 0.001). This was not observed in the IUGR group (ρ = − 0.149; *p* = 0.306) (Fig. 1a). When cholesterol concentrations were related to birth weights ((cholesterol (mmol/L)) / (birth weight (g))), no significant difference was detected between IUGR and CTRL fetuses in all gestational weeks at birth (Fig. 1b). Assuming that the fetal serum cholesterol concentration (mmol/L) mirrors fetal tissue cholesterol content (mmol/kg), the fetal body accumulates twice as much cholesterol during intrauterine development (calculated as cholesterol (mmol/L) * birth weight (kg)) in CTRL as in IUGR (Fig. 1c).

### Cholesterol synthesis

**Fig. 2:**
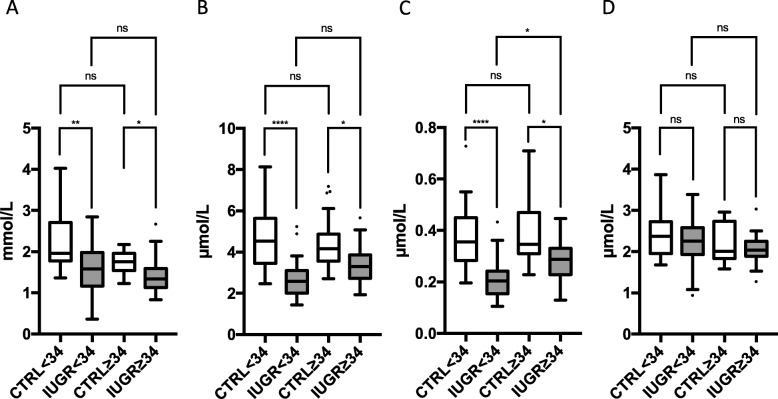
Fetal concentration of **a** cholesterol, **b** lathosterol, **c** lanosterol, and **d** desmosterol in umbilical cord blood of early onset (< 34 WOG, *n* = 24) and late onset (≥34 WOG, *n* = 25) IUGR fetuses compared to a healthy control group with adequate birth weight and born at similar gestational age (< 34 WOG, *n* = 25; ≥34 WOG, *n* = 24). Tukey’s box plots. Dots represent outliers. ns “not significant”; *p < 0.05; **p < 0.01; ***p < 0.001; ****p < 0.0001

In IUGR, absolute lanosterol and lathosterol concentrations were 35 and 33% lower than in CTRL (Table 2). Adjustment for potential confounders had no major influence on the significance. The lanosterol/TC and lathosterol/TC ratios did not differ significantly (Table 3). We found no significant difference in mean desmosterol concentrations between both study groups (Table 2), although in the IUGR group, the desmosterol/TC ratio was 30% higher than in the CTRL group (Table 3). Findings were similar for the two subgroups, early onset and late onset IUGR (Fig. 2), but we failed to provide significance for desmosterol/TC ratio in the late onset IUGR group compared to their gestational age matched CTRL group (data not shown).

**Table 2:** Total sterol concentrations in umbilical cord vein of IUGR and CTRL neonates

Total concentration		CTRL (*n* = 49)		IUGR (*n* = 49)		*p*-value	adj. *p*-value
		Median	95% CI	Median	95% CI		
Cholesterol (GCMS)	mmol/L	1.90800	(1.75800–1.96900)	1.44600	(1.28500–1.61000)	**< 0.0001**	**< 0.0001**
Lathosterol	μmol/L	4.34600	(3.87600–4.69100)	2.97500	(2.67200–3.24700)	**< 0.0001**	**0.0009**
Dihydro-lanosterol	μmol/L	0.01248	(0.01009–0.01462)	0.00806	(0.00652–0.01101)	**0.0019**	**0.0040**
Desmosterol	μmol/L	2.20600	(1.98800–2.50300)	2.13100	(2.01400–2.25800)	0.3145	0.8708
Lanosterol	μmol/L	0.34760	(0.31850–0.41090)	0.23700	(0.21380–0.27620)	**< 0.0001**	**0.0001**
24OH cholesterol	μmol/L	0.14230	(0.12250–0.15840)	0.14870	(0.13130–0.16870)	0.6355	0.8430
27OH cholesterol	μmol/L	0.06026	(0.05529–0.06517)	0.06440	(0.05743–0.07782)	0.2345	0.3010
β-sitosterol	μmol/L	0.78970	(0.61960–0.94940)	0.66460	(0.56740–0.82710)	0.2451	0.3916
Campesterol	μmol/L	0.73540	(0.63070–0.82030)	0.62460	(0.56650–0.76840)	0.5196	0.3462
5α-cholestanol	μmol/L	10.66000	(10.15000–11.75000)	9.72100	(9.13700–10.47000)	**0.0061**	**0.0159**
Stigmasterol	μmol/L	0.13850	(0.13220–0.16650)	0.11290	(0.10060–0.14100)	**0.0011**	**0.0060**
Brassicasterol	μmol/L	0.08838	(0.07519–0.10950)	0.08332	(0.07112–0.10850)	0.9605	0.3390

Significant *p*-values are in bold

**Table 3:** Ratios of specific sterol to cholesterol concentrations in umbilical cord vein of IUGR and CTRL neonates

		CTRL (*n* = 49)		IUGR (*n* = 49)		*p*-value
Sterol to TC ratio	μmol/mmol *10exp3	Median	95% CI	Median	95% CI	
Lathosterol/TC	Ratio	2.183	(2.049–2.523)	2.081	(1.894–2.276)	0.2952
Dihydro-lanosterol/TC	Ratio	0.006	(0.005–0.008)	0.006	(0.005–0.008)	0.6267
Desmosterol/TC	Ratio	1.156	(1.080–1.259)	1.522	(1.399–1.586)	**< 0.0001**
Lanosterol/TC	Ratio	0.187	(0.167–0.206)	0.175	(0.145–0.206)	0.1795
24OH cholesterol/TC	Ratio	0.075	(0.065–0.084)	0.108	(0.091–0.123)	**< 0.0001**
27OH cholesterol/TC	Ratio	0.033	(0.030–0.035)	0.047	(0.043–0.050)	**< 0.0001**
β-sitosterol/TC	Ratio	0.420	(0.354–0.481)	0.478	(0.418–0.558)	**0.0210**
Campesterol/TC	Ratio	0.387	(0.360–0.429)	0.469	(0.437–0.569)	**0.0002**
5α-cholestanol/TC	Ratio	5.990	(5.426–6.489)	6.924	(6.515–7.175)	**0.0004**
Stigmasterol/TC	Ratio	0.075	(0.069–0.085)	0.082	(0.070–0.094)	0.1585
Brassicasterol/TC	Ratio	0.045	(0.040–0.053)	0.059	(0.055–0.066)	**0.0001**

Significant *p*-values are in bold

### Oxysterols

**Fig. 3:**
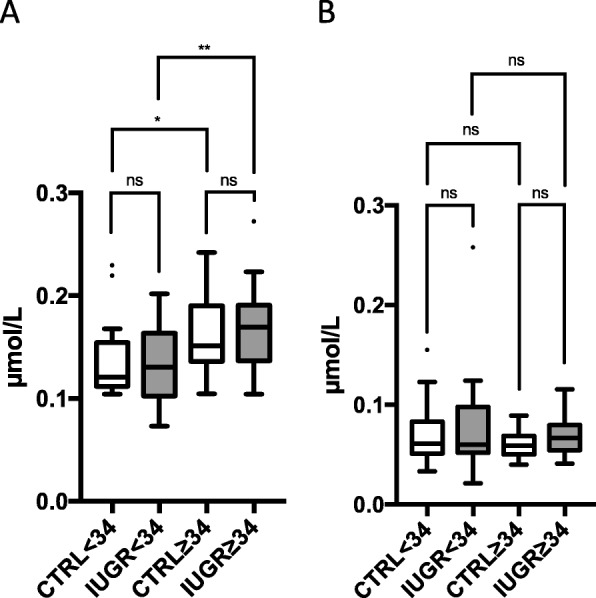
Fetal concentration of **a** 24-hydroxy-cholesterol and **b** 27-hydroxy-cholesterol in umbilical cord blood of early onset (< 34 WOG, *n* = 24) and late onset (≥34 WOG, *n* = 25) IUGR fetuses compared to a healthy control group with adequate birth weight and born at similar gestational age (< 34 WOG, *n* = 25; ≥34 WOG, *n* = 24). Tukey’s box plots. Dots represent outliers. ns “not significant”; *p < 0.05; **p < 0.01; ***p < 0.001; ****p < 0.0001

No significant differences were found in absolute 24S-OH-cholesterol (24S-OHC) and 27-OHC levels in IUGR compared to CTRL (Table 2). Adjustment for potential confounders had no major influence on significance. Hence, 24S-OHC/TC ratio and 27-OHC/TC ratio were increased by 39 and 61%, respectively, indicating an increased cholesterol turnover (Table 3). Findings were similar in the early and the late onset subgroups of IUGR (Fig. 3).

### Plant sterols

**Fig. 4:**
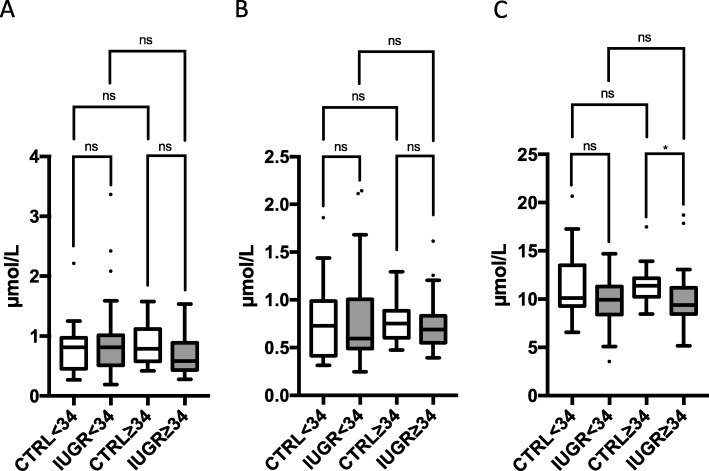
Fetal concentration of **a** β-sitosterol, **b** campesterol, and **c** 5α-cholestanol in umbilical cord blood of early onset (< 34 WOG, *n* = 24) and late onset (≥34 WOG, *n* = 25) IUGR fetuses compared to a healthy control group with adequate birth weight and born at similar gestational age (< 34 WOG, *n* = 25; ≥34 WOG, *n* = 24). Tukey’s box plots. Dots represent outliers. ns “not significant”; *p < 0.05; **p < 0.01; ***p < 0.001; ****p < 0.0001

No significant differences were found in absolute campesterol and β-sitosterol levels, while in the IUGR group, 5α-cholestanol levels were in the mean 13% lower than in the CTRL group (Table 2). Adjustment for potential confounders had no major influence on significance. Ratios of campesterol, β-sitosterol, and 5α-cholestanol to TC were increased by 34, 39, and 17%, respectively (Table 3). However, this could not be confirmed when separately analyzing data in the late onset subgroup of IUGR (Fig. 4).

## Discussion

Several studies, including our own, have reported diminished fetal cholesterol concentrations in IUGR [17–23]. Characterizing umbilical cord blood sterol profiles in IUGR and normally grown fetuses we aimed at a deeper understanding of cholesterol metabolism during fetal development and impaired fetal growth. To estimate the contribution of maternal cholesterol to the fetal cholesterol pool, we measured plant sterol concentrations such as β-sitosterol. Plant sterols cannot be synthesized de novo in humans. Their concentration levels in the circulation are dependent on nutritional intake, and, in the case of the fetus, maternal provision. At term, concentration levels of plant sterols in umbilical cord blood are 40–50% of the maternal levels [27]. Consequently, placental insufficiency and disturbed placental transport processes should result in lower fetal plant sterol concentrations. By contrast to this assumption, plant sterol to cholesterol ratios were indeed higher in IUGR than in CTRL fetuses, especially in those born preterm, suggesting an unrestricted transplacental sterol transport and a general substantial maternal contribution to the fetal cholesterol pool independent of placental insufficiency. High plant sterol to cholesterol ratios can, however, also be explained by lower hepato-biliary elimination rates of the phytosterols. The liver is the main organ regulating body cholesterol homeostasis. The synthesis of cholesterol starts with the mevalonate pathway by the formation of acetyl CoA, which is then converted to 3-hydroxy-3-methylglutaryl CoA (HMG-CoA) and further to mevalonate, squalene and finally, lanosterol. Lanosterol can be processed to cholesterol through an enzyme-mediated 19-step process following the Kandutsch-Russell pathway through lathosterol and 7-dehydrocholesterol. In the present study, low circulating lanosterol and lathosterol concentrations together with balanced cholesterol ratios suggest an unaffected enzymatic capacity of the Kandutsch-Russell pathway in IUGR. Hence, reduced lanosterol concentration in IUGR is most probably caused by lower enzymatic activity prior to the mevalonate pathway, or by a decrease in nutritional supply or energy provision to the fetal liver. Indeed, in IUGR, a reduced placental oxygen uptake and glucose transfer forces the fetus to increase shunting through the ductus venosus bypassing the liver to maintain nutritional supply to the heart and the brain [33, 34]. This so-called “brain-sparing-effect” results in a reduced hepatic volume [35–37], declining hepatic glycogen stores, and fetal hypoglycemia, and affects maintenance of fetal oxidative metabolism [20, 34, 38]. As a result, diminished synthesis of acetyl-CoA in the fetal liver and, subsequently, reduced mevalonate and lanosterol production can be expected.

Interestingly, and in contrast to lathosterol, the desmosterol to cholesterol ratios in our study were higher in the IUGR than in the CTRL group. Desmosterol is synthesized from lanosterol via zymosterol. A high ratio suggests either increased synthesis rates or reduced conversion to cholesterol via the 3β-hydroxysterol-Δ24-reductase (DHCR24), the final enzymatic step of the Bloch pathway, an alternative pathway of cholesterol synthesis [30]. The Bloch pathway is particularly activated in the fetal brain and desmosterol transiently represents up to 30% of total brain sterols during intrauterine development [24, 39]. Desmosterol might cross from fetal brain into the body circulation either via an incomplete blood-brain barrier (BBB), or via to date unknown pathways, e.g. potential re-conversion of brain-driven 24S-OHC to desmosterol. While the latter enzymatic step is theoretically possible, a specific enzyme has yet to be described. However, the presence of cholesterol transport molecules in the BBB suggests a potential flux of sterols from the brain into the body circulation [40, 41]. This is particularly important since fetal brain cholesterol homeostasis must be tightly regulated, separately from the body cholesterol homeostasis for beneficial neurodevelopment. Almost all inborn errors of cholesterol synthesis are associated with central neural system malformations or neuro-developmental disorders [15, 42]. In line with desmosterol, 24S-OHC to cholesterol ratios were evenly high in the IUGR fetuses in our study. 24S-OHC is a cholesterol metabolite almost exclusively produced in the brain that crosses the BBB to protect from cholesterol overload [43, 44]. In light of the “brain-sparing-effect”, this finding of desmosterol and 24S-OHC suggests conserved brain cholesterol turnover rates in IUGR fetuses independent of body cholesterol synthesis to guarantee uneventful neurodevelopment.

Since cholesterol and its metabolites are catabolized in the fetal liver by bile acid formation higher 24S-OHC/TC ratios (and also 27-OHC/TC ratios) in IUGR fetuses may also be explained by reduced elimination rates. Indeed, studies on liver function of IUGR fetuses suggest a reduced hepatic activity with lower alanine aminotransferase synthesis rates [45], and higher bile acids concentrations in cord blood of the IUGR fetuses as compared to controls [46] that persists in childhood [47].

These persisting metabolic alterations in IUGR neonates as well as increased plant sterol concentrations are in concert with the known observation that being small for gestational age is a risk factor for CVD later in life. High plasma plant sterol levels have been frequently associated with CVD [48]. Campesterol/cholesterol and sitosterol/cholesterol ratios, e.g., have been demonstrated to be independent predictors of CVD [49]. Mechanistically, plant sterols promote production of proinflammatory cytokines, influence ABC transporter expression, and inhibit cholesterol efflux from macrophages [50]. Indeed, we recently demonstrated a lower cholesterol efflux from [^3^H]-cholesterol-loaded macrophages when treated with serum from IUGR compared to CTRL fetuses [51] possibly contributing to early atherosclerotic changes of the fetal vascular walls.

The present study has certain limitations. IUGR fetuses often have to be delivered preterm, and gestational age is one of the main factors affecting cord blood composition [17]. Hence, the study groups need to be controlled for this issue. However, preterm birth per se is a pathological condition and we cannot exclude that cord blood parameters within the CTRL group are biased by factors that may lead to spontaneous onset of preterm labor. Moreover, differences in the treatment regimens between the IUGR and the CTRL mothers may have an influence on maternal and/or fetal metabolism. For example, mothers with spontaneous onset of preterm labor or preterm premature rupture of membranes receive antibiotics and tocolytic agents, while IUGR mothers do not. Stress also influences lipid parameters, and fetal distress is a common medical indication for delivery in patients with IUGR. We also do not have data on nutritional intake of patients, which may influence plant sterol concentrations.

Strength of this study is its well characterized patient cohort and antenatal diagnosis and clear definition of “IUGR”. The use of GC methods resulted in a highly specific assessment of the sterols of interest, avoiding the frequently observed cross-reactivity that occurs with conventional antibody-based measurement techniques.

## Conclusion

We here provide evidence that in IUGR fetuses, the fetal de novo cholesterol synthesis rate rather than the materno-fetal cholesterol transfer is low. Our data suggest that reduced energy provision forces the IUGR fetus to downregulate hepatic de novo cholesterol synthesis, yet this seems to be rather adaptive and well balanced to meet fetal growth rates. Additionally, reduced hepatic function causes lower elimination rates of sterols and cholesterol end-products. This observation is particularly important, since cholestasis is a well-described complication in extremely low birth weight neonates [52] that could progress to liver failure [53] and may affect hepatic drug metabolism [54]. Our study supports the idea that specific therapeutic regimes need to be considered in neonates born IUGR, especially in the setting of neonatal intensive care management. This applies to drug administration, but also to parenteral nutrition of the neonate with plant sterol containing soybean oil-based lipid emulsions.

## Data Availability

The datasets used and/or analyzed during the current study are available from the corresponding author upon reasonable request.

## Abbreviations

24S-OHC: 24S-OH-cholesterol
27-OHC: 27-OH-cholesterol
BBB: Blood-brain barrier
BMI: Body mass index
CTRL: Controls
CVD: Cardiovascular diseases
DHCR24: 3β-hydroxysterol-Δ24-reductase
FID: Flame-ionization detection
GC: Gas chromatographic
HMG-CoA: 3-hydroxy-3-methylglutaryl CoA
IUGR: Intrauterine growth restriction
MS-SIM: Selected-ion monitoring mass spectrometry
SLOS: Smith-Lemli-Opitz syndrome
TC: Total cholesterol
TG: Triglycerides
WOG: Week of gestation

## References

[1] ACOG (2013). ACOG Practice bulletin no. 134: fetal growth restriction. Obstet Gynecol.

[2] Kehl S, Dötsch J, Hecher K, Schlembach D, Schmitz D, Stepan H (2017). Intrauterine growth restriction. Guideline of the German Society of Gynecology and Obstetrics (S2k-level, AWMF registry no. 015/080, October 2016). Geburtshilfe Frauenheilkd.

[3] Barker DJ (1995). Fetal origins of coronary heart disease. BMJ..

[4] Forsen T, Eriksson JG, Tuomilehto J, Osmond C, Barker DJ (1999). Growth in utero and during childhood among women who develop coronary heart disease: longitudinal study. BMJ..

[5] Kaijser M, Bonamy AK, Akre O, Cnattingius S, Granath F, Norman M (2008). Perinatal risk factors for ischemic heart disease: disentangling the roles of birth weight and preterm birth. Circulation..

[6] Rich-Edwards JW, Kleinman K, Michels KB, Stampfer MJ, Manson JE, Rexrode KM (2005). Longitudinal study of birth weight and adult body mass index in predicting risk of coronary heart disease and stroke in women. BMJ..

[7] Biri A, Bozkurt N, Turp A, Kavutcu M, Himmetoglu O, Durak I (2007). Role of oxidative stress in intrauterine growth restriction. Gynecol Obs Invest.

[8] Burton GJ, Woods AW, Jauniaux E, Kingdom JC (2009). Rheological and physiological consequences of conversion of the maternal spiral arteries for uteroplacental blood flow during human pregnancy. Placenta..

[9] Pecks U, Rath W, Caspers R, Sosnowsky K, Ziems B, H-JH-J T (2013). Oxidatively modified LDL particles in the human placenta in early and late onset intrauterine growth restriction. Placenta..

[10] Pecks U, Rath W, Kleine-Eggebrecht N, Maass N, Voigt F, Goecke TW (2016). Maternal serum lipid, estradiol, and progesterone levels in pregnancy, and the impact of placental and hepatic pathologies. Geburtshilfe Frauenheilkd.

[11] Baardman ME, Erwich JJ, Berger RM, Hofstra RM, Kerstjens-Frederikse WS, Lutjohann D (2012). The origin of fetal sterols in second-trimester amniotic fluid: endogenous synthesis or maternal-fetal transport?. Am J Obs Gynecol..

[12] Björkhem I, Diczfalusy U, Lütjohann D (1999). Removal of cholesterol from extrahepatic sources by oxidative mechanisms. Curr Opin Lipidol.

[13] Björkhem I, Eggertsen G (2001). Genes involved in initial steps of bile acid synthesis. Curr Opin Lipidol.

[14] Shackleton CHL (2012). Role of a disordered steroid metabolome in the elucidation of sterol and steroid biosynthesis. Lipids..

[15] Kanungo S, Soares N, He M, Steiner RD (2013). Sterol metabolism disorders and neurodevelopment-an update. Dev Disabil Res Rev.

[16] Jenkins KT, Merkens LS, Tubb MR, Myatt L, Davidson WS, Steiner RD (2008). Enhanced placental cholesterol efflux by fetal HDL in smith-Lemli-Opitz syndrome. Mol Genet Metab.

[17] Pecks U, Brieger M, Schiessl B, Bauerschlag DOO, Piroth D, Bruno B (2012). Maternal and fetal cord blood lipids in intrauterine growth restriction. J Perinat Med.

[18] Merzouk H, Meghelli-Bouchenak M, El-Korso N, Belleville J, Prost J (1998). Low birth weight at term impairs cord serum lipoprotein compositions and concentrations. Eur J Pediatr.

[19] Spencer JA, Chang TC, Crook D, Proudler A, Felton CV, Robson SC (1997). Third trimester fetal growth and measures of carbohydrate and lipid metabolism in umbilical venous blood at term. Arch Dis Child Fetal Neonatal Ed.

[20] Bon C, Raudrant D, Poloce F, Champion F, Golfier F, Pichot J (2007). Biochemical profile of fetal blood sampled by cordocentesis in 35 pregnancies complicated by growth retardation. Pathol Biol.

[21] Roberts A, Nava S, Bocconi L, Salmona S, Nicolini U (1999). Liver function tests and glucose and lipid metabolism in growth-restricted fetuses. Obstet Gynecol.

[22] Nagano N, Okada T, Fukamachi R, Yoshikawa K, Munakata S, Usukura Y (2013). Insulin-like growth factor-1 and lipoprotein profile in cord blood of preterm small for gestational age infants. J Dev Orig Health Dis.

[23] Pecks U, Rath W, Maass N, Berger B, Lueg I, Farrokh A (2016). Fetal gender and gestational age differentially affect PCSK9 levels in intrauterine growth restriction. Lipids Health Dis.

[24] Lin DS, Pitkin RM, Connor WE (1977). Placental transfer of cholesterol into the human fetus. Am J Obs Gynecol.

[25] Burke KT, Colvin PL, Myatt L, Graf GA, Schroeder F, Woollett LA (2009). Transport of maternal cholesterol to the fetus is affected by maternal plasma cholesterol concentrations in the golden Syrian hamster. J Lipid Res.

[26] Pecks U, Mohaupt MG, Hütten MC, Maass N, Rath W, Escher G (2014). Cholesterol acceptor capacity is preserved by different mechanisms in preterm and term fetuses. Biochim Biophys Acta - Mol Cell Biol Lipids.

[27] Vuorio AF, Miettinen TA, Turtola H, Oksanen H, Gylling H (2002). Cholesterol metabolism in normal and heterozygous familial hypercholesterolemic newborns. J Lab Clin Med.

[28] Tranquilli AL, Dekker G, Magee L, Roberts J, Sibai BM, Steyn W (2014). The classification, diagnosis and management of the hypertensive disorders of pregnancy: a revised statement from the ISSHP. Pregnancy Hypertens.

[29] Voigt M, Rochow N, Schneider KTM, Hagenah H-P, Scholz R, Hesse V (2014). New percentile values for the anthropometric dimensions of singleton neonates: analysis of perinatal survey data of 2007-2011 from all 16 states of Germany. Z Geburtshilfe Neonatol.

[30] Lütjohann D, Brzezinka A, Barth E, Abramowski D, Staufenbiel M, von Bergmann K (2002). Profile of cholesterol-related sterols in aged amyloid precursor protein transgenic mouse brain. J Lipid Res.

[31] Taubert A, Silva LMR, Velásquez ZD, Larrazabal C, Lütjohann D, Hermosilla C (2018). Modulation of cholesterol-related sterols during Eimeria bovis macromeront formation and impact of selected oxysterols on parasite development. Mol Biochem Parasitol.

[32] Mackay DS, Jones PJH, Myrie SB, Plat J, Lütjohann D (2014). Methodological considerations for the harmonization of non-cholesterol sterol bio-analysis. J Chromatogr B Analyt Technol Biomed Life Sci.

[33] Kiserud T, Kessler J, Ebbing C, Rasmussen S (2006). Ductus venosus shunting in growth-restricted fetuses and the effect of umbilical circulatory compromise. Ultrasound Obstet Gynecol.

[34] Baschat AA (2004). Fetal responses to placental insufficiency: an update. BJOG..

[35] Flood K, Unterscheider J, Daly S, Geary MP, Kennelly MM, McAuliffe FM (2014). The role of brain sparing in the prediction of adverse outcomes in intrauterine growth restriction: results of the multicenter PORTO study. Am J Obstet Gynecol.

[36] Campbell S, Thoms A (1977). Ultrasound measurement of the fetal head to abdomen circumference ratio in the assessment of growth retardation. Br J Obs Gynaecol.

[37] Molina Giraldo S, Alfonso Ayala DA, Arreaza Graterol M, Perez Olivo JL, Solano Montero AF (2019). Three-dimensional Doppler ultrasonography for the assessment of fetal liver vascularization in fetuses with intrauterine growth restriction. Int J Gynaecol Obstet.

[38] Lane RH, Kelley DE, Gruetzmacher EM, Devaskar SU (2001). Uteroplacental insufficiency alters hepatic fatty acid-metabolizing enzymes in juvenile and adult rats. Am J Physiol Regul Integr Comp Physiol.

[39] Jansen M, Wang W, Greco D, Bellenchi GC, di Porzio U, Brown AJ (2013). What dictates the accumulation of desmosterol in the developing brain?. FASEB J.

[40] Gosselet F, Candela P, Sevin E, Berezowski V, Cecchelli R, Fenart L (2009). Transcriptional profiles of receptors and transporters involved in brain cholesterol homeostasis at the blood-brain barrier: use of an in vitro model. Brain Res.

[41] Kober AC, Manavalan APC, Tam-Amersdorfer C, Holmér A, Saeed A, Fanaee-Danesh E (2017). Implications of cerebrovascular ATP-binding cassette transporter G1 (ABCG1) and apolipoprotein M in cholesterol transport at the blood-brain barrier. Biochim Biophys Acta.

[42] Virgintino D, Errede M, Girolamo F, Capobianco C, Robertson D, Vimercati A (2008). Fetal blood-brain barrier P-glycoprotein contributes to brain protection during human development. J Neuropathol Exp Neurol.

[43] Björkhem I, Lütjohann D, Diczfalusy U, Ståhle L, Ahlborg G, Wahren J (1998). Cholesterol homeostasis in human brain: turnover of 24S-hydroxycholesterol and evidence for a cerebral origin of most of this oxysterol in the circulation. J Lipid Res.

[44] Lütjohann D, Breuer O, Ahlborg G, Nennesmo I, Sidén A, Diczfalusy U (1996). Cholesterol homeostasis in human brain: evidence for an age-dependent flux of 24S-hydroxycholesterol from the brain into the circulation. Proc Natl Acad Sci U S A.

[45] Kocylowski R, Dubiel M, Gudmundsson S, Fritzer E, Kiserud T, von Kaisenberg C (2012). Hepatic aminotransferases of normal and IUGR fetuses in cord blood at birth. Early Hum Dev.

[46] Boehm G, Müller DM, Teichmann B, Krumbiegel P (1990). Influence of intrauterine growth retardation on parameters of liver function in low birth weight infants. Eur J Pediatr.

[47] Sidiropoulou EJ, Paltoglou G, Valsamakis G, Margeli A, Mantzou A, Papassotiriou I (2018). Biochemistry, hormones and adipocytokines in prepubertal children born with IUGR evoke metabolic, hepatic and renal derangements. Sci Rep.

[48] Vergès B, Fumeron F (2015). Potential risks associated with increased plasma plant-sterol levels. Diabetes Metab.

[49] Rajaratnam RA, Gylling H, Miettinen TA (2000). Independent association of serum squalene and noncholesterol sterols with coronary artery disease in postmenopausal women. J Am Coll Cardiol.

[50] Sabeva NS, McPhaul CM, Li X, Cory TJ, Feola DJ, Graf GA (2011). Phytosterols differentially influence ABC transporter expression, cholesterol efflux and inflammatory cytokine secretion in macrophage foam cells. J Nutr Biochem.

[51] Pecks U, Rath W, Bauerschlag DO, Maass N, Orlikowsky T, Mohaupt MG, et al. Serum Cholesterol Acceptor Capacity in Intrauterine Growth Restricted Fetuses. J Perinat Med. 2017; accepted. DOI 10.1515/jpm-2016-0270. 10.1515/jpm-2016-0270 28195552

[52] Kelly DA (1998). Liver complications of pediatric parenteral nutrition--epidemiology. Nutrition..

[53] Zambrano E, El-Hennawy M, Ehrenkranz RA, Zelterman D, Reyes-Múgica M (2004). Total parenteral nutrition induced liver pathology: an autopsy series of 24 newborn cases. Pediatr Dev Pathol.

[54] Soo JY, Wiese MD, Berry MJ, McMillen IC, Morrison JL (2018). Intrauterine growth restriction may reduce hepatic drug metabolism in the early neonatal period. Pharmacol Res.

